# Canine Endometrial Mesenchymal Stem Cells: Characterization and Functional Assessment for Cartilage Repair

**DOI:** 10.3390/ijms26168091

**Published:** 2025-08-21

**Authors:** Zuzana Vikartovska, Marcela Maloveska, Natalia Nosalova, Lubica Hornakova, Mykhailo Huniadi, Nikola Hudakova, Slavomir Hornak, Blazej Kalinaj, Peter Kubatka, Dasa Cizkova

**Affiliations:** 1 Small Animal Clinic, Centre of Experimental and Clinical Regenerative Medicine, University of Veterinary Medicine and Pharmacy in Kosice, Komenskeho 73, 041 81 Kosice, Slovakia; zuzana.vikartovska@uvlf.sk (Z.V.); marcela.maloveska@gmail.com (M.M.); natalia.nosalova@uvlf.sk (N.N.); lubica.hornakova@uvlf.sk (L.H.); mykhailo.huniadi@uvlf.sk (M.H.); nikola.hudakova@uvlf.sk (N.H.); slavomir.hornak@uvlf.sk (S.H.); blazej.kalinaj@student.uvlf.sk (B.K.); peter.kubatka@uvlf.sk (P.K.); 2 Institute of Parasitology, Slovak Academy of Sciences, Hlinkova 3, 040 01 Kosice, Slovakia; 3 Department of Morphological Disciplines, University of Veterinary Medicine and Pharmacy in Kosice, Komenskeho 73, 041 81 Kosice, Slovakia

**Keywords:** canine endometrial mesenchymal stem cells, conditioned medium, chondrocyte metabolic activity, oxidative stress, cartilage repair, regenerative medicine

## Abstract

Endometrial mesenchymal stem cells (eMSCs) are a novel and biologically potent source of multipotent stromal cells with potential beyond reproductive medicine. This study explored their phenotypic profile, trilineage differentiation, and the cytoprotective effects of their conditioned media (eMSCCM) on oxidatively stressed neonatal and adult chondrocytes. Canine eMSCs displayed typical fibroblast-like morphology and expressed high levels of mesenchymal surface markers CD29 and CD44, low hematopoietic markers CD34/CD45, and variable CD90, confirming a mesenchymal identity. Differentiation assays revealed osteogenic and chondrogenic differentiation, whereas adipogenic activity was limited. Using eMSCCM at 25% and 50% concentrations, chondrocyte viability was assessed after exposure to 200 µM H_2_O_2_. eMSCCM significantly enhanced the viability of H_2_O_2_-stressed chondrocytes in a dose-dependent manner, particularly at 50%, with marked effects at 24 and 48 h. Although metabolic activity declined at 72 h, the treated cells remained more metabolically active than untreated controls. These findings suggest that eMSCCM offers promising cytoprotective effects for cartilage-related oxidative stress conditions.

## 1. Introduction

In recent years, regenerative veterinary medicine has focused on stem cell-based and cell-free therapies for repairing damaged tissues. Among the most promising strategies are mesenchymal stem cells (MSCs) and their conditioned media (CM) enriched with extracellular vesicles (EVs), which are recognized for their availability, straightforward extraction process, capacity to promote tissue repair, immunomodulatory properties, and multipotent differentiation potential [1]. Although MSCs are commonly obtained from bone marrow, adipose tissue, or the umbilical cord, emerging evidence suggests that the endometrium is a promising and underexplored source of stem/progenitor cells [2]. This potential is supported by the periodic remodeling of the endometrium during reproductive life, which suggests that endometrial-derived stem cells may play a crucial role in tissue regeneration and repair [3]. Various stem and progenitor cell types have been identified and studied from human endometrial tissues, including human endometrial mesenchymal stem cells (heMSCs), endometrial epithelial progenitors (eEP), and side population (SP) cells with regenerative abilities [3,4]. Among them, heMSCs exhibit unique biological properties, such as secreting cytokines, trophic factors, and EVs, that stimulate angiogenesis, immunomodulation, and anti-inflammatory effects [4,5].

Similar to the human endometrium, the canine endometrium naturally regenerates, allowing for the isolation of canine endometrial-derived mesenchymal stem cells (eMSCs) with strong paracrine activity and high proliferative potential [6]. Based on their biological properties, they can be utilized in veterinary medicine to treat uterine pathologies, reproductive problems, as well as systemic degenerative diseases unrelated to the reproductive system [4,5].

Focal cartilage lesions, particularly chondral and osteochondral defects, represent the most common and clinically challenging conditions affecting joint function in companion animals [7]. Due to the limited intrinsic regenerative capacity of articular cartilage, these defects often lead to progressive joint degeneration. Emerging research supports MSC-based therapies for cartilage repair in dogs, demonstrating advantages such as reduced pain and discomfort, improved lameness, and the formation of hyaline-like cartilage [8,9]. However, considerable variability exists in the methodologies, cell sources, and application strategies employed across studies, limiting direct comparisons and standardization [8]. Most studies to date have focused on MSCs from more traditional sources (bone marrow, adipose tissue, synovium) for chondral defect repair [10]. In contrast, eMSCs are relatively novel in this context, but their comparable paracrine profile suggests they may be a promising candidate for cartilage repair.

Despite their encouraging therapeutic potential, standardized protocols for the collection, comprehensive characterizations, and functional analyses of eMSCs and their conditioned media (eMSCCM) are still lacking [3,11].

The objectives of this study are to develop a standardized isolation procedure and to define the biological properties of eMSCs, including their capacity for differentiation and proliferation; furthermore, to evaluate the cytoprotective efficacy of secreted eMSCCM on stressed neonatal and adult chondrocytes, a crucial step toward their potential therapeutic application in veterinary regenerative medicine. By offering an in-depth evaluation of these cells and their ability to promote tissue repair, we could propose the therapeutic application of eMSCs into veterinary regenerative medicine, particularly in the context of personalized therapeutic applications for cartilage defects, aligning with the principles of precision and predictive medicine.

## 2. Results

### 2.1. Morphology of eMSCs

**Figure 1:**
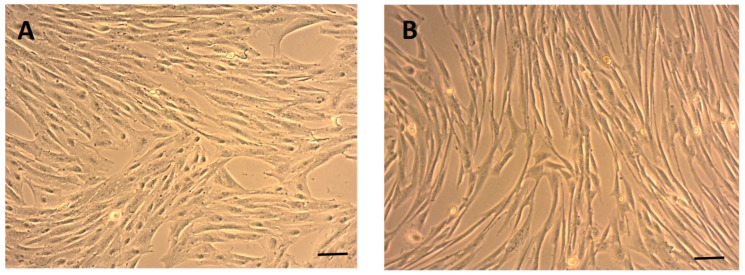
Morphology of eMSCs after passaging. Phase-contrast images show eMSCs exhibiting typical fibroblast-like, spindle-shaped morphology under standard culture conditions, passage 0 (P0), DIV7 (**A**), with active growth, revealing a uniform monolayer with elongated cytoplasmic extensions, P3, DIV10 (**B**), characteristic of mesenchymal stem cells. Scale bars: 50 µm.

The eMSCs quickly attached to tissue culture plastic surfaces within 24 h, revealing small, elongated cells with either a bipolar or multipolar shape at 7 days in vitro (DIV7) (Figure 1A). As they continued to grow, they retained consistent morphology up to passage 3 (P3) (Figure 1B), suggesting a stable phenotype important for further applications in regenerative medicine.

### 2.2. Multilineage Differentiation

**Figure 2:**
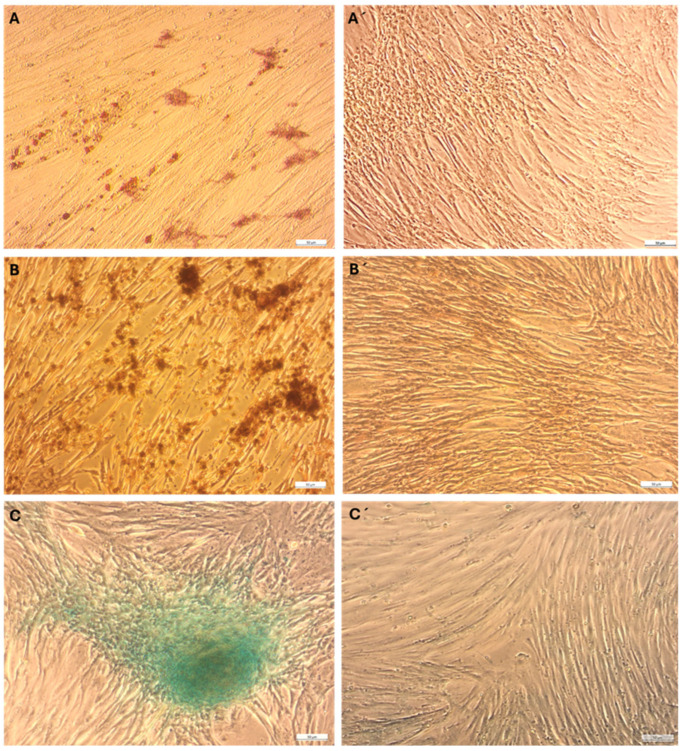
Trilineage differentiation of eMSCs. Representative images showing differentiation into adipogenic (**A**), osteogenic (**B**), and chondrogenic (**C**) lineages alongside respective negative controls (**A′**–**C′**). Adipogenic differentiation, confirmed by Oil Red O staining, was revealed by the rare presence of intracellular lipid droplets (**A**). Osteogenic differentiation was visualized by Alizarin Red S staining, indicating calcium deposition (**B**). Micromass formation and Alcian Blue-positive staining of extracellular sulfated glycosaminoglycans indicated chondrogenic differentiation (**C**). Scale bars = 50 µm.

Adipogenic Differentiation—eMSCs grown in adipogenic differentiation medium occasionally exhibited a rounded morphology with intracellular lipid vacuoles, as detected by Oil Red O staining. However, these features were rare indicators of adipogenic differentiation at 15 days of differentiation (Figure 2A).

Osteogenic Differentiation—Osteogenic differentiation was induced by culturing eMSCs in a medium supplemented with osteoinductive factors, including ascorbic acid, β-glycerophosphate, and dexamethasone. Alizarin Red S staining after 18 days revealed calcium deposition in the extracellular matrix, indicating osteogenic differentiation (Figure 2B).

Chondrogenic Differentiation—Chondrogenic differentiation was evaluated through the micromass culture technique to develop three-dimensional chondrogenic aggregates. The eMSCs cultured in chondrogenic differentiation medium maintained spherical cell aggregates that resembled early-stage cartilage structures. These aggregates displayed significant Alcian Blue staining, which specifically binds to glycosaminoglycans (GAGs), a key component of the cartilage extracellular matrix (Figure 2C). These results illustrate the tripotent potential of eMSCs, consistent with mesenchymal stem cell identity.

### 2.3. Flow Cytometry Analysis of eMSC Surface Marker Expression

**Figure 3:**
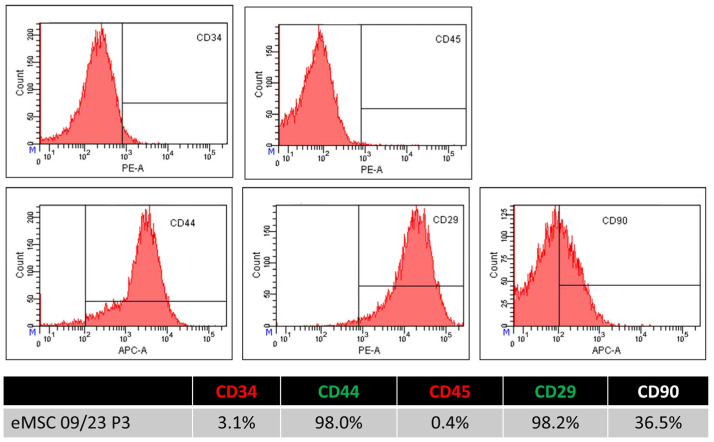
Flow cytometry analysis of surface marker expression in eMSCs. Histograms represent the expression of eMSC CD29, CD44, and CD90, and low expression of hematopoietic markers CD34 and CD45. High expression of CD29 and CD44 confirms the mesenchymal phenotype, while the low expression of CD34 and CD45 indicates minimal contamination by hematopoietic cells.

To assess the immunophenotype of eMSCs, flow cytometry analysis was conducted using antibodies against eMSCs markers CD29, CD44, and CD90, alongside hematopoietic markers CD34 and CD45. Cells were stained with fluorophore-conjugated antibodies and analyzed on a BD FACSCanto™ cytometer. Gating was performed to exclude debris, dead cells, and doublets, and data were analyzed using BD FACS DivaTM software, version 6.1.3. The eMSCs showed robust expression of CD29 (average: 98.13% ± 0.27%) and CD44 (96.30% ± 1.41%), with consistently high levels across all replicates, confirming their mesenchymal identity. CD90 expression was more variable, with a mean of 17.70% ± 14.86%, indicating the presence of a distinct subpopulation or potential heterogeneity at this passage. In contrast, the cells were negative for hematopoietic markers CD34 (2.80% ± 2.38%) and CD45 (2.97% ± 2.20%), consistent with the expected phenotype of mesenchymal rather than hematopoietic origin. These findings support the mesenchymal phenotype of eMSCs, particularly through high CD29 and CD44 expression, while also highlighting the variability in CD90, which may reflect donor-specific or culture-dependent differences (Figure 3), Table 1.

### 2.4. XTT Assay

**Figure 4:**
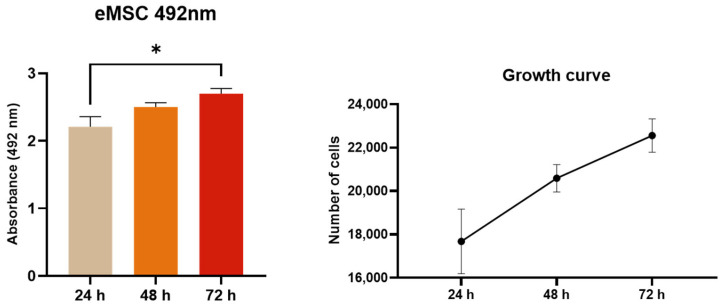
Metabolic activity of eMSCs assessed by XTT assay over time. The graph shows the optical density (OD) values measured at 492 nm after 24, 48, and 72 h of incubation. A time-dependent increase in absorbance indicates enhanced mitochondrial activity and cell proliferation under standard culture conditions. Data are presented as mean ± standard deviation (SD) from (*n* = 4). Statistical significance was determined by one-way ANOVA, with * *p* < 0.05 considered significant.

The metabolic activity of eMSCs was evaluated using the XTT assay. Absorbance increased significantly at the 72-h time point in comparison to the 24-h time point, suggesting the time-dependent metabolic activity of eMSCs under standard culture conditions. The growth curve revealed dynamic growth of cell number over the studied time (Figure 4).

### 2.5. Morphology, Alcian Blue Staining, and Collagen II Expression of Primary Chondrocytes

**Figure 5:**
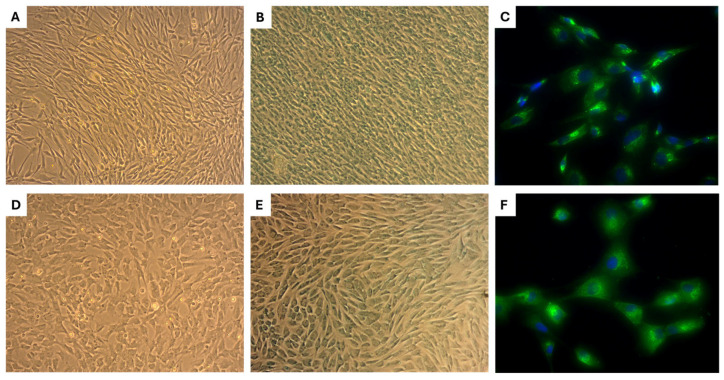
Morphology and chondrogenic features of primary chondrocytes. Neonatal chondrocytes exhibited a long, bipolar morphology (**A**), while adult chondrocytes displayed a polygonal shape (**D**). Alcian blue staining revealed strong positivity in both cell types (**B**,**E**). Immunofluorescent staining for type II collagen (COL II) showed cytoplasmic localization and extension into cellular processes for both chondrocyte populations (**C**,**F**). Nuclei were counterstained with DAPI (blue) (**C**,**F**). Scale bars: (**A**,**B**,**D**,**E**) = 100×; (**C**,**F**) = 400×.

Chondrocytes derived from neonatal cartilage exhibited a smaller size and predominantly bipolar morphology, in contrast to chondrocytes derived from adult cartilage, which were larger and displayed a polygonal shape (Figure 5A,D). Alcian Blue staining demonstrated comparable metachromatic staining intensity in both neonatal and adult chondrocyte cultures, indicating the presence of sulfated glycosaminoglycans (Figure 5B,E). Immunofluorescence analysis confirmed the expression of type II collagen (COL II), which was primarily localized within the cytoplasm of chondrocytes, suggesting active-matrix protein synthesis (Figure 5C,F). These findings confirm that both neonatal and adult chondrocyte cultures isolated from canine cartilages maintain a chondrogenic phenotype and are suitable for functional in vitro studies involving stress induction and endometrium-derived mesenchymal stem cell–conditioned medium (eMSCCM) treatment.

### 2.6. Evaluation of Cytoprotective Properties of eMSCCM on Stressed Chondrocytes

**Figure 6:**
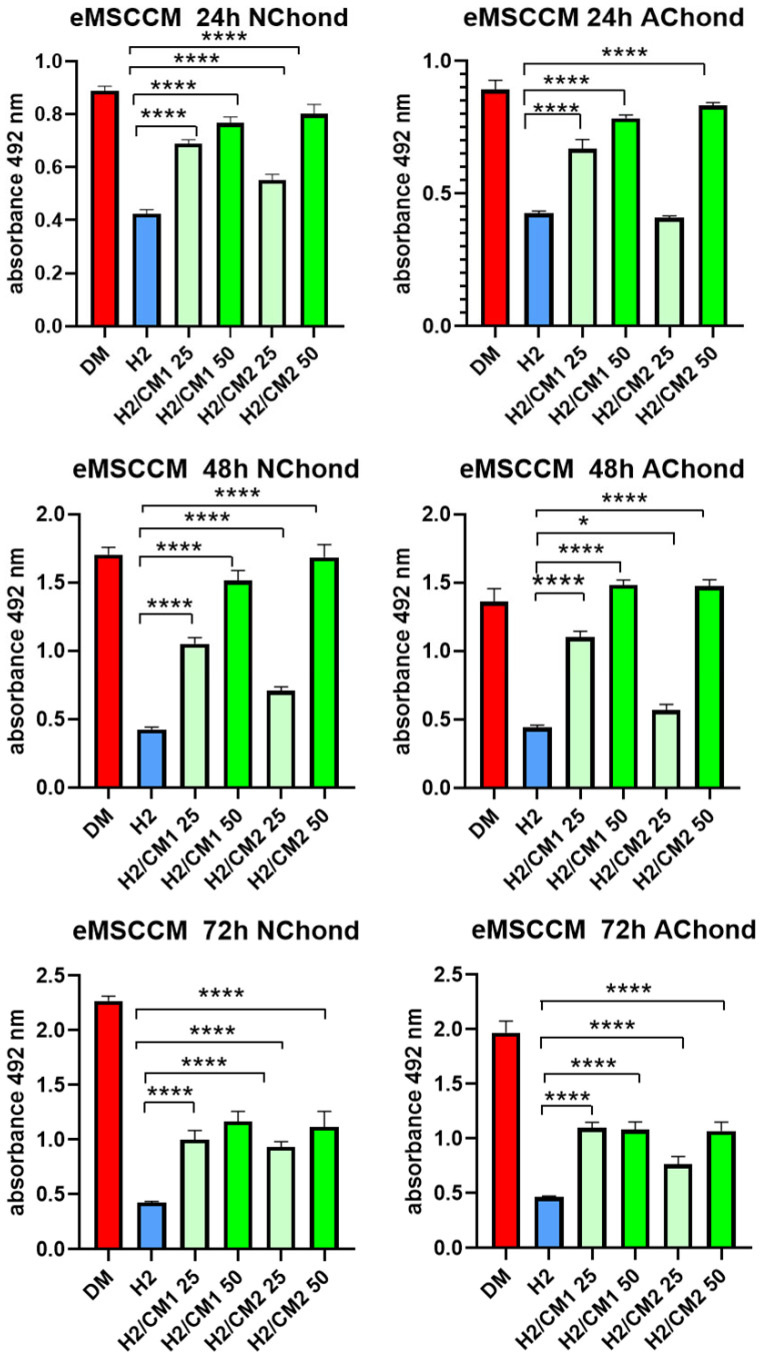
Cytoprotective effect of eMSCCM on oxidatively stressed chondrocytes over time. Primary neonatal (NChond) and adult (AChond) chondrocytes were cultured in standard DMEM (DM), or exposed to oxidative stress (200 µM H_2_O_2_; H2), and treated with two independently prepared eMSCCM pools (CM1 and CM2) at concentrations of 25% and 50%. Cell viability was assessed at 24 h, 48 h, and 72 h post-treatment. Both CM1 and CM2 exhibited cytoprotective effects, with greater variability observed at the 25% concentration between the two pools, particularly at earlier time points. At 50%, both CM pools consistently restored chondrocyte viability across all time intervals. The most pronounced cytoprotective effects were detected at 24 and 48 h, while at 72 h, metabolic activity decreased, though it remained significantly higher than in the untreated group (H2). Data are presented as mean ± SD (*n* = 5). Statistical significance was determined using one-way ANOVA; * *p* < 0.05, **** *p* < 0.0001 was considered significant, compared to the stressed untreated group H2.

To assess the cytoprotective potential of eMSCCM, oxidatively stressed neonatal (NChond) and adult chondrocytes (AChond) were treated with two independently prepared CM pools (CM1 and CM2) at concentrations of 25% and 50%. As expected, exposure to 200 µM H_2_O_2_ (H2) significantly reduced chondrocyte viability. Treatment with both CM1 and CM2 restored metabolic activity in a concentration-dependent manner. However, at the lower 25% concentration, variability in the cytoprotective effect was observed between CM1 and CM2, suggesting inter-pool differences in bioactivity at diluted levels. In contrast, at the 50% concentration, both CM1 and CM2 consistently improved chondrocyte viability, with no significant differences between the pools, indicating a more robust and reproducible effect at this higher concentration. The strongest cytoprotective effects were observed at 24 and 48 h, while at 72 h the metabolic activity declined, though it remained significantly higher than in the untreated H2 group. These findings confirm the beneficial effects of eMSCCM and underscore the importance of concentration in minimizing variability between CM batches isolated from 12 donors (CM1 pool/1–6 donors, CM2pool/7–12 donors) (Figure 6).

## 3. Discussion

This study provides a comprehensive and mechanistically grounded characterization of canine eMSCs and demonstrates the cytoprotective efficacy of their conditioned medium (eMSCCM, secretome) on oxidatively injured chondrocytes. According to the obtained findings, the eMSCCM shows therapeutic potential for application in regenerative veterinary medicine, particularly for cartilage repair.

Following isolation, eMSCs rapidly adhered to tissue culture plastic flasks and displayed a fibroblast-like morphology across passages 3–5, indicating a phenotypically stable and proliferative population. This is critical, as MSCs are documented to undergo continuous functional decline and replicative senescence as early as the first passage, including telomere shortening, gene expression drift, and reduced differentiation potential, which impede clinical efficacy. Ensuring early passage stability is thus essential for reliable therapeutic application [12,13,14,15,16].

The trilineage differentiation assays further confirmed the multipotent nature of eMSCs. While adipogenic differentiation was limited, possibly due to endometrial origin and hormone-responsive epigenetic signature of these cells [17], the osteogenic and chondrogenic potential was clearly demonstrated. Osteogenic commitment was evident, consistent with previous reports using similar osteoinductive factors [17,18]. Chondrogenic differentiation, assessed using micromass culture, recapitulates early cartilage development and highlights the applicability of eMSCs in cartilage tissue engineering [19].

On the other hand, the weak adipogenic response seen in our study could be caused by both internal and external factors. First of all, the dynamic, hormone-responsive environment from which endometrial MSCs originate is not favorable for lipid storage or adipocyte lineage commitment [20]. The transcriptional program and epigenetic landscape of eMSCs have been suggested to navigate them to mesodermal lineages other than adipocytes, particularly chondrocytes and osteoblasts [17]. Furthermore, eMSCs have been shown to exhibit fewer adipogenic regulators, such as PPARγ and C/EBPα, which are essential for adipocyte development [21]. Finally, culture conditions and induction methods, particularly using commercialized kits, might not be entirely beneficial for eMSCs, highlighting the need for tissue-specific adipogenic media optimization with custom protocols [22,23]. In this context, it has been proposed that adipogenic conversion in hormonally responsive MSCs can be improved by co-stimulation with estrogen modulators, 3D culture, or hypoxic preconditioning.

Flow cytometric analysis confirmed the mesenchymal identity of the isolated cells, showing strong and consistent expression of reference MSC markers CD29 and CD44 and a lack of hematopoietic markers CD34 and CD45 [12]. However, variability in CD90 expression occurred, similar to that reported by others [24]. This may reflect donor-related heterogeneity or culture-induced subpopulation differences [11,15]. The immunophenotype of endometrial resident stem cells, including the expression of surface markers like CD90, can be influenced by tissue-specific microenvironmental cues, such as the endometrium’s dynamic hormonal milieu [25,26]. Furthermore, it has been shown that MSCs from a variety of sources exhibit epigenetic regulation and post-transcriptional modulation of CD90 expression, especially while expanding in vitro or under stress [27,28]. CD90 expression may change as part of a temporary phenotypic plasticity associated with immunomodulatory activation or the differentiation status of the cells [29].

A hypoxic environment can sustain or even increase CD90 expression [30]. These findings imply that CD90 variability should be understood as a context-sensitive phenotypic response, potentially reflecting stemness maintenance, immunomodulatory activation, or lineage priming, rather than a loss of multipotency [20].

In summary, although we did not perform quantitative marker-based profiling to determine the exact percentage of MSCs, their trilineage differentiation capacity, along with the expression of specific CD markers (≥98%), remain a key functional hallmark of MSCs’ identity. This aligns with the established ISCT criteria and the emerging veterinary-specific standards proposed for MSCs [12,31]. Although the characterization of canine MSCs remains limited, ongoing research is expected to identify more specific and reliable combinations of surface markers to improve their definition and standardization [31]. However, it should be considered that other cell populations may be co-isolated in variable and often unknown proportions when deriving MSCs from canine endometrium [3,4]. This limitation is consistent with previous findings in the field and reflects a common challenge associated with MSC isolation from complex, heterogeneous tissue environments [32]. Furthermore, some degree of biological variability between donor-derived preparations cannot be excluded despite standardized isolation procedures [33].

In the study’s second section, we used both neonatal and adult chondrocytes to assess the functional cytoprotective ability of eMSCCM in an oxidative stress paradigm. Involving well-documented models of oxidative cartilage damage and the established function of reactive oxygen species (ROS) in osteoarthritic degeneration, exposure to 200 µM H_2_O_2_ markedly reduced chondrocyte viability [28,34]. Oxidative stress disrupts redox homeostasis, leading to mitochondrial dysfunction, lipid peroxidation, DNA fragmentation, and apoptotic signaling in chondrocytes, events implicated in cartilage matrix degradation and disease progression [35].

The bioactivity of the secreted molecules was confirmed by the concentration-dependent restoration of chondrocyte metabolic activity following treatment with eMSCCM. Two independently generated pools (CM1 and CM2) were prepared by pooling media from donors CM1–CM6 and from donors CM7–CM12. This approach allowed us to assess the overall secretory profile of eMSC-derived CM across all 12 donors. These pools showed varying cytoprotective effects at 25% concentration, whereas at 50%, both pools enhanced cell metabolic activity. According to earlier publications demonstrating inter-pool heterogeneity in MSC-derived products, these discrepancies are probably caused by variances in donor cells, culture conditions, or processing procedures [32]. These results highlight how crucial it is to standardize CM preparation procedures in order to guarantee uniformity and repeatability in therapeutic applications [5]. Higher concentrations may buffer batch-to-batch variability by delivering sufficient amounts of bioactive molecules, as both CM pools consistently increased chondrocyte survival at the higher concentration of 50% across all periods. While metabolic activity remained much higher than the untreated stress group (H2), the greatest cytoprotective benefits were shown at 24 and 48 h, with a slight drop at 72 h. This decrease may be the result of oxidative damage that has not been completely restored by CM or the deterioration of active molecules. In addition, our findings did not reveal any significant differences in vulnerability to oxidative stress or treatment between neonatal and adult chondrocytes. The observed variations were solely dose-dependent.

Current literature includes only a limited number of studies investigating the protective effects of CM derived from endometrial MSCs, particularly those obtained from menstrual blood (MenSCs). One notable study demonstrated the beneficial effects of such CM in a Parkinson’s disease model, likely due to the presence of trophic factors [36].

Beyond neuroprotection, other studies have reported immunomodulatory properties of endometrial stem cell-derived CM. CM from non-endometriosis-derived MenSCs (NE-MenSCs) was able to normalize gene expression in endometriosis-derived endometrial stem cells (E-MenSCs), specifically targeting genes associated with inflammation and stemness [37]. Additionally, regenerative CM from endometrial sources significantly alleviated clinical symptoms and improved histological outcomes in a mouse model of colitis [38].

Taken together, these findings underscore the broad therapeutic potential of endometrial stem cell-derived CM across neurological, inflammatory, and reproductive contexts. Future research should focus on elucidating its cytoprotective mechanisms, which are likely driven by a synergistic combination of soluble bioactive factors.

These may include hepatocyte growth factor (HGF), insulin-like growth factor-1 (IGF-1), transforming growth factor-β (TGF-β), and IL-10, as well as bioactive EVs, including exosomes enriched with antioxidant enzymes, miRNAs (e.g., miR-21, miR-140), and heat-shock proteins [39,40]. MSCs-derived EVs have been recently shown to modulate oxidative stress by promoting the expression of antioxidant genes (e.g., SOD2, GPX1, and HO-1), inhibiting pro-apoptotic pathways (e.g., Bax/Bcl-2 ratio, caspase-3 activity), and preserving mitochondrial membrane potential [41]. These mechanisms are especially relevant for chondrocyte survival in inflammatory or degenerative conditions, where ROS generation perpetuates extracellular matrix (ECM) breakdown and inflammation [42]. However, oxidative stress-induced senescence, which alters MSC secretory activity and promotes paracrine senescence in neighboring cells, must be considered when evaluating the therapeutic benefits of MSC-based therapy, but not MSCCM, which already contains secreted factors [43].

Our findings extend the therapeutic potential of eMSCs and their secretome beyond disorders of the reproductive system. This observation is particularly significant for tissues with limited regenerative capacity, such as articular cartilage. Importantly, we demonstrated that the conditioned medium derived from an easily accessible tissue, such as from uterus, routinely available during elective ovariohysterectomy (OVH), can influence the regeneration of completely unrelated tissues in the same individual. This concept opens new perspectives in personalized regenerative medicine, where autologous cell populations and their bioactive secretome, including extracellular vesicles, could be harvested, cryopreserved, and later used for the treatment of both reproductive and non-reproductive pathologies in the donor. In a time when standard therapies often fail, particularly in degenerative and chronic diseases, there is an urgent need to exploit endogenous cellular sources that are frequently discarded as biological waste. Utilizing their untapped regenerative potential offers a sustainable and ethically acceptable treatment strategy. Our work contributes a small yet meaningful piece to the broader mosaic of regenerative medicine, a rapidly evolving field that is poised to become a defining force of future medical practice.

Collectively, these results show that eMSCs reveal a persistent, distinct mesenchymal phenotype with strong differentiation potential toward chondrogenic and osteogenic lineages. With applicability in models of osteoarthritis and cartilage damage, their secretome offers a repeatable, cell-free method of shielding cartilage cells from oxidative stress. These findings are consistent with data that MSC secretomes can be used in cell-free regenerative therapy in both veterinary and human medicine [42]. To translate these insights into clinical practice, future efforts must focus on: (a) optimizing CM production, including media composition, preconditioning, and delivery systems (e.g., hydrogels for sustained release); (b) profiling active components such as cytokines, antioxidants, heat-shock proteins, and EV cargo via Nrf2/HO-1 and other pathways; (c) rigorous in vivo validation to assess therapeutic efficacy, dosage response, and biosafety; (d) establishing standardized quality control frameworks to support regulatory approval and clinical scalability.

## 4. Materials and Methods

### 4.1. Animal Selection and Ethical Approval

A total of 12 healthy female dogs, aged 1 to 5 years and weighing 10–30 kg, were selected. All procedures were conducted with the informed consent of the owners at the University Veterinary Hospital, UVLF Košice. The protocol for MSC isolation from biological waste was approved by the Ethics Committee of the UVLF Košice (approval numbers EKVP/2022-21 and EKVP/2025-3).

### 4.2. Hormonal Status Assessment: Anesthesia and Surgical

Blood samples were collected from the *vena cephalica antebrachii* using tubes with agglutination gel. Samples were centrifuged at 2050× *g* for 10 min (Eppendorf 5720M centrifuge, Darmstadt, Germany). The progesterone level was determined using the Speed Progesterone™ Test (Virbac, France), a fluorescence immunoassay, analyzed with the Speed Reader™ analyzer. Progesterone levels were measured on the day of surgery, and all animals showed levels below 1 ng/mL, confirming they were in the anoestrus period.

### 4.3. Anesthesia and Surgery

Sedation was initiated with butorphanol (BUTOMIDOR™ Richter Pharma AG, Wels, Austria, 0.2 mg/kg IV) and medetomidine (CEPETOR™ KH, CP-Pharma, Burgdorf, Germany, 0.015–0.02 mg/kg IV). Propofol (PROPOFOL™ MCT/LCT Fresenius 10 mg/mL, Fresenius Kabi, Friedberg, Germany, 2–3 mg/kg IV) was used to induce anesthesia, as needed, for intubation. Anesthesia was maintained with sevoflurane (SEVORANEvap inl, AbbVie, Campoverde di Aprilia, Italy, 2–2.5%) in oxygen via inhalation using a rebreathing anaesthetic machine (Dräger Vapor 2000 Sevoflurane, Dräger Fabius Tiro™, Lübeck, Germany). Throughout the procedure, vital parameters—heart rate, respiratory rate, oxygen saturation, and end-tidal CO_2_ were continuously monitored. Under general anesthesia, celiotomy was performed and the uterus exteriorized. Ovaries were double ligated and excised, followed by removal of uterine horns via double ligation (circular and penetrating) above the cervix. Antibiotics, NSAIDs, and fluid therapy were administered as supportive care during the operation. The abdominal wall was closed using a standard multilayer technique.

### 4.4. Sample Collection

**Figure 7:**
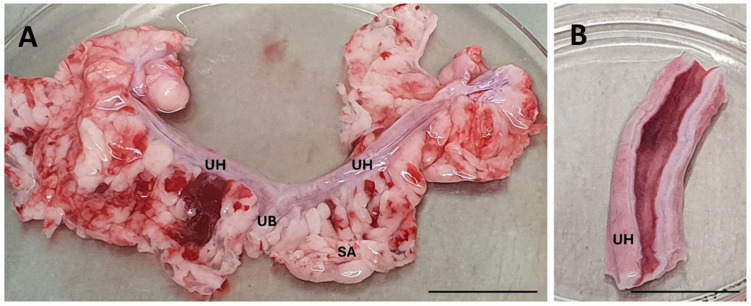
Dissected canine uterus separated from the reproductive system, note the two elongated uterine horns (UH) connected to a short uterine body (UB) with suspensory apparatus (SA) (**A**). The uterine horn was opened longitudinally to expose the endometrial lining used for the isolation of eMSCs (**B**). Scale bars = 4.5 cm.

Ovaries, uterine tubes, uterus, and the suspensory apparatus (classified as biological waste) were collected aseptically. All biological material was immediately placed in cold (4 °C) sterile phosphate-buffered saline (PBS, Sigma-Aldrich, St. Louis, MO, USA). Samples were processed within one hour of collection to preserve the viability and structure of cells for downstream analysis (see Figure 7A,B).

### 4.5. Isolation of eMSCs

The uterus was separated from the reproductive system, placed in a sterile Petri plate, and rinsed with PBS containing 2% antibiotic-antimycotic (ATB/ATM, Sigma-Aldrich, St. Louis, MO, USA). The uterine horns were opened longitudinally, exposing the endometrial lining. Tissue samples were collected from the middle section of the uterine horn (Figure 7B). Two grams of tissue were mechanically separated using a scalpel and enzymatically digested with 0.05% Collagenase IV (Gibco, Waltham, MA, USA) at 37 °C for 30 min. The resulting suspension was filtered through a 100 µm sterile nylon mesh filter (Fisher Scientific^®^, Waltham, MA, USA) to remove undigested debris. The filtered fraction was centrifuged at 300× *g* for 8 min and the pellet was resuspended in Dulbecco’s Modified Eagle Medium/Nutrient Mixture F-12 (DMEM-F12, Sigma-Aldrich, St. Louis, MO, USA) supplemented with 10% fetal bovine serum (FBS, Sigma-Aldrich, St. Louis, MO, USA), 2% ATB/ATM, and 1% L-glutamine (Biowest, Nuaillé, France). Cells were seeded in T25 culture flasks (Corning Inc., Corning, NY, USA) at a density of 1 × 10^6^ cells/mL and incubated at 37 °C with 5% CO_2_. Non-adherent cells were removed, and the medium was changed twice weekly.

### 4.6. Cell Passaging

Cells were passaged upon reaching 75–80% confluence using 0.25% Trypsin-EDTA (Biowest, Nuaillé, France) for 5–7 min at 37 °C. Trypsin activity was neutralized with 10% FBS. Cells were centrifuged at 300× *g* for 8 min, resuspended in fresh culture medium, and transferred to T75 culture flasks for expansion. During the experimental study, cells from passages 1 to 4 were used.

### 4.7. Morphology and Metabolic Activity of eMSCs

Phase-contrast microscopy was used to evaluate morphology. Cellular metabolic activity was quantified using the XTT cell proliferation Kit II (Roche Diagnostics, Mannheim, Germany). Cells from passage 3 were seeded at 2 × 10^3^ cells/well in a 96-well plate (Corning Inc., Corning, NY, USA). Standard cell concentrations of 1 × 10^3^, 2 × 10^3^, 3 × 10^3^, 4 × 10^3^, and 5 × 10^3^ cells/well were used in the cultivation medium as a control. Optical density was measured at 492 nm after 24, 48, and 72 h using a spectrophotometer the APOLLO Absorbance Reader (Berthold Systems, Oak Ridge, TN, USA). The number of cells was calculated using a previously established calibration curve correlating optical density to cell count. The growth curve was then plotted by representing the cell number over time, allowing the assessment of proliferation dynamics. The following formula, N = OD492 − b/m, was used to calculate the number of cells, where N is the number of cells, OD492 is the measured optical density, and m and b are constants obtained from the calibration curve’s linear regression.

### 4.8. Multilineage Differentiation

To evaluate differentiation into osteogenic, adipogenic, and chondrogenic lineages, cells at passage 3 were plated in 24-well plates (Corning Inc., Corning, NY, USA). For the adipogenic and osteogenic differentiation assays, cells were seeded at a density of 5 × 10^4^ cells per well. Chondrogenic differentiation was initiated by placing three 5 µL micromasses containing 6 × 10^4^ cells in the center of each well, followed by a 2-h incubation at 37 °C with 5% CO_2_. Afterward, the cultivation media with appropriate supplements from the Commercial StemPro Differentiation Kits (Thermo Fisher Scientific, Waltham, MA, USA) were added according to the manufacturer’s instructions. The cultures were then fixed with 4% formaldehyde and stained with Oil Red O (for adipogenesis), Alizarin Red (for osteogenesis), and Alcian Blue (for chondrogenesis) to confirm multilineage differentiation.

### 4.9. Flow Cytometry

The eMSCs at passage 3, revealing 80–90% confluency, were rinsed with PBS to eliminate residual FBS, and adherent cells were detached using 0.25% trypsin-EDTA (Thermo Fisher Scientific, Waltham, MA, USA) for 3–5 min at 37 °C. The released cell suspension was then transferred into 15 mL conical tubes and centrifuged at 250× *g* for 5 min at room temperature. The cell pellet was resuspended in 1 mL of PBS; the cells were counted using a trypan blue exclusion assay, and a minimum of 2 × 10^5^ cells per sample/100 µL were prepared for staining. The cells were labeled with the following fluorophore-conjugated monoclonal antibodies to identify surface hematopoietic markers: CD34-PE (phycoerythrin), CD45-PE, and mesenchymal stem cell markers CD44-APC (allophycocyanin), CD29-PE, and CD90-APC (all from Sigma-Aldrich, St. Louis, MO, USA). To each 100 µL PBS containing 2 × 10^5^ cells, 1–2 µL of each conjugated antibody was added. The samples were gently mixed and then incubated in the dark at room temperature for 45 min to avoid photobleaching of the fluorophores; afterward, the cells were rinsed twice with PBS and centrifuged at 250× *g* for 5 min. Lastly, the cell pellets were resuspended in 100 µL of PBS and kept on ice in the dark until they were analyzed. Samples were evaluated using a BD FACSCanto™ flow cytometer (Becton Dickinson Biosciences, San Jose, CA, USA), with three lasers (488 nm, 633 nm, and 405 nm). To obtain results, at least 10,000 events for each sample were recorded. The presence or absence of marker expression was confirmed using unstained controls.

### 4.10. Isolation of Conditioned Medium from Endometrial MSC (eMSCCM)

Conditioned medium (CM) from eMSCCM was obtained following the protocol described in our previous study [44]. Briefly, eMSCs at passage P3 were cultured in DMEM-HG supplemented with 10% FBS and 1% ATB/ATM. The cells were seeded at a density of 1.2 × 10^6^ per T75 culture flask. The medium was removed after reaching approximately 80% confluence (48–72 h), and the cells were rinsed twice with sterile PBS to remove any remaining FBS. Subsequently, each flask was supplemented with 5 mL of FBS-free DMEM-LG with 1% ATB/ATM. The cells were then cultured under standard conditions (37 °C, 5% CO_2_) to promote the release of bioactive molecules into the medium. After 24 h, the eMSCCM (*n* = 12) were collected, centrifuged to eliminate cell debris, and stored at −80 °C for later use in downstream functional experiments.

### 4.11. Primary Cultures of Canine Chondrocytes

In our in vitro experiments, we used neonatal chondrocytes, whose isolation and detailed characterization were described extensively in our recent work [45]. Additionally, in the current study, we included adult chondrocytes, which were isolated from femoral head samples obtained during orthopedic procedures (*n* = 2, German Shepherd), using an identical isolation protocol as for neonatal samples [45]. All samples were used for tissue dissection following the acquisition of informed consent signed by the owners. The isolation procedure was conducted with the approval of the Ethics Committee of the UVLF Kosice (EKVP/2025-3). The aim was to compare potential differences in sensitivity to oxidative stress between neonatal and adult chondrocytes, as well as to evaluate possible variations in therapeutic response to eMSCCM collected from different individuals (CM1–12).

### 4.12. Histochemical and Immunocytochemical Staining of Chondrocytes

Neonatal and adult chondrocytes (P3) were seeded in monolayer cultures (2500 cells/cm^2^ in 24-well plates or 1 × 10^4^ cells/well on Lab-Tek chamber slides) and maintained for one week in DMEM with 10% FBS and 1% ATB/ATM. For glycosaminoglycan detection, cells were fixed with 4% paraformaldehyde (PFA) and stained with 1% Alcian Blue for 30 min, followed by washes with 0.1 N HCl and distilled water (all from Sigma-Aldrich, St. Louis, MO, USA). For immunocytochemistry, cells were permeabilized with PBS containing 10% NGS and 0.1% Triton X-100, then incubated overnight at 4 °C with anti-collagen II antibody (1:100). After washing, FITC-conjugated secondary antibody (1:250) and DAPI (1:250) were applied (all from Abcam, Waltham, MA, USA). Fluorescence imaging was performed using a Zeiss Axio Observer Z1 microscope with Apotome 3 and FS81 filter cube (DAPI-FITC) (Carl Zeiss AG, Oberkochen, Germany) to confirm COL II expression.

### 4.13. Functional In Vitro Analyses: Cytoprotective Effect of eMSCCM on Stressed Chondrocytes

#### 4.13.1. Oxidative Stress Induction in Primary Canine Chondrocyte Cultures

For the functional analyses of eMSCCM, we employed the oxidative stress model previously characterized in our study [45]. To ensure representative and consistent results, we prepared two pooled samples of endometrial eMSCCM from a total of 12 donor dogs. Specifically, eMSCCM1 was created by pooling conditioned media from six donors (CM1–CM6), and eMSCCM2 by pooling CM from another six donors (CM7–CM12). This approach allowed us to evaluate the overall secretory profile of eMSCs while minimizing individual donor variability. We compared the cytoprotective effects of eMSCCM1 (pooled 1–6 CM samples) and eMSCCM2 (pooled 7–12 CM samples) on chondrocytes exposed to oxidative stress induced by hydrogen peroxide (H_2_O_2_).

#### 4.13.2. XTT Assay

Neonatal and adult chondrocytes (passage 2) were seeded into 96-well plates at a density of 10 × 10^3^ cells per well in 100 μL of DMEM supplemented with 1% ATB/ATM and 5% FBS. The cells were allowed to adhere and grow for 24 h. After a 24 h incubation in DMEM, the cells were either incubated in DMEM (control) or exposed to H_2_O_2_ at concentrations of 200 μM (H2) for 60 min. Following H_2_O_2_ treatment, the cells were washed with Dulbecco’s phosphate buffer saline (DPBS, Gibco, Waltham, MA, USA USA) to remove residual H_2_O_2_. The cells were then incubated with eMSCCM1 (pool CM1–6) diluted in DMEM (CM1 25%, CM1 50% in DMEM), and with eMSCCM2 (pool CM7–12) diluted in DMEM (CM2 25%, CM2 50% in DMEM). The treatment effect of CM1 and CM2 on the H_2_O_2_-stressed chondrocytes was assessed after 24, 48, and 72 h of incubation under standard in vitro conditions using XTT assay (Roche, Grenzach-Wyhlen, Germany) performed in quintuplicates (*n* = 5) for each condition. A fresh XTT solution was prepared by mixing 5 mL of XTT labeling reagent with 0.1 mL of electron coupling reagent. A volume of 50 μL of the XTT mixture was added to each well, and the plates were incubated for 4 h at 37 °C with 5% CO_2_. Absorbance was measured at 492 nm using an AMR-100 Microplate reader (Hangzhou Allsheng Instruments Co., Ltd., Zhuantang Town, China) to determine cell metabolic activity.

## 5. Conclusions

Canine eMSCs maintain a stable mesenchymal phenotype and demonstrate strong osteogenic and chondrogenic differentiation capacities. Their conditioned medium consistently provides dose-dependent, cytoprotective effects against oxidative stress in chondrocytes. The data support the development of standardized, cell-free secretome therapies for cartilage repair in clinical practice, offering a scalable alternative to cell transplantation. Future directions should involve refinement of CM production protocols, detailed profiling of bioactive components and EVs cargo, and in vivo efficacy and safety studies to translate these findings into regenerative veterinary and translational therapies.

## Figures and Tables

**Table 1:** The table summarizes the expression variability of each CD marker along with mean values and standard deviations.

Passage 3	CD34	CD45	CD29	CD44	CD90
eMSC 01	0.00	5.30	97.80	96.00	11.60
eMSC 02	5.30	3.20	98.40	94.90	5.00
eMSC 03	3.10	0.40	98.20	98.00	36.50
Average	2.80	2.97	98.13	96.30	17.70
STDEV	2.381	2.198	0.273	1.405	14.858

## Data Availability

The raw data supporting the conclusions of this article will be made available by the authors upon reasonable request.
